# Potent neutralization of Rift Valley fever virus mediated by monoclonal antibodies via concurrent inhibition of attachment and fusion

**DOI:** 10.1080/22221751.2026.2623698

**Published:** 2026-02-10

**Authors:** Meng Hao, Ting Bian, Zhengshan Chen, Guanying Zhang, Chuanyi Zhao, Guangcheng Fu, Yi Chen, Xiangyang Chi, Pengfei Fan, Ting Fang, Changming Yu, Jianmin Li

**Affiliations:** a Laboratory of Advanced Biotechnology, Beijing Institute of Biotechnology, Beijing, People’s Republic of China; b Laboratory of Advanced Biotechnology, ZJU-Hangzhou Global Scientific and Technological Innovation Center, Hangzhou, People’s Republic of China; c Jiangsu Academy of Agricultural Sciences, Institute of Veterinary Medicine, Nanjing, People’s Republic of China

**Keywords:** RVFV, neutralizing antibodies, Gn protein, antiviral mechanism, attachment and fusion inhibition

## Abstract

Rift Valley fever virus (RVFV) is one of the most important mosquito-borne pathogens that causes substantial morbidity and mortality in livestock and humans. Despite its public health and economic impact, no licenced vaccines or therapeutics are currently available for human use. Here, we report the isolation of a panel of Gn-specific monoclonal antibodies (mAbs) from the memory B cells of rhesus macaques immunized with Ad4-GnGc or Ad5-GnGc. 20 mAbs with neutralizing activity were identified and divided into two groups, targeting subdomain I and subdomain III of the Gn protein, respectively. In murine infection models, representative nAbs A38 and A13 demonstrated efficacious protection against RVFV infection in both prophylactic and therapeutic settings. Research on the neutralizing mechanisms of antibodies revealed that A13 mainly mediates neutralization by inhibiting RVFV fusion to cells, while A38 disrupts multiple stages of the viral entry process by blocking both virus attachment and membrane fusion. To gain deeper insights into these mechanisms, we predicted the variable regions of antibodies and performed molecular docking with RVFV Gn head domain. Structural analysis showed that A38 binds to the DI and DIII subdomains, while A13 binds to an epitope spanning three subdomains of Gn, likely preventing the structural rearrangements required for membrane fusion. This study identifies multiple promising therapeutic candidates against RVFV and elucidates the structural mechanisms by which neutralizing antibodies inhibit various stages of the viral life cycle. These findings deepen our understanding of RVFV pathogenesis and will facilitate the development of novel therapeutic strategies.

## Introduction

Rift Valley fever virus (RVFV), the causative agent of Rift Valley fever (RVF), is a mosquito-borne haemorrhagic fever virus which poses a significant threat to both human and animal health [1]. First identified in 1931 during a fatal outbreak among sheep in Kenya's Rift Valley [2], the virus has since expanded its geographic range beyond the African continent. Outbreaks have been reported in regions such as Madagascar, Saudi Arabia, Yemen, and the Comoros Islands [3,4]. In 2016, China documented its first imported case of RVF in a traveller returning from Angola, a non-endemic country [5]. The growing mobility of human populations and livestock trade, driven by economic globalization, alongside climate-driven shifts in mosquito and reservoir species distribution, and the widespread presence of susceptible hosts and many other ecological factors, has significantly increased the risk of RVF spreading to new geographic regions [6-8]. RVFV causes severe disease in livestock, especially sheep and goats, leading to high rates of mortality in newborn lambs and abortion storms in ewes, dramatically impacting the socio-economic situation in endemic areas [9-11]. Human infected with RVFV often display a self-limiting febrile illness, while less than 5% of infected individuals may progress to neurological disorders, lethal haemorrhagic fever, or blindness [12]. Despite extensive efforts to develop vaccines and antiviral agents since its discovery, no RVFV-specific vaccines or therapies have yet been licenced for human use. This gap highlights the critical need for the development of effective antiviral treatments to address the ongoing threat posed by the virus.

RVFV is a negative-sense RNA virus with envelope belonging to the *Bunyavirales* order and *Phenuiviridae* family [13]. This virus has a tripartite genome consisting of three genome segments: S (small), M (medium), and L (large). The M segment of the RVFV genome encodes two glycoproteins, Gn and Gc, which are crucial for viral attachment, entry, and fusion [14,15]. Gn forms the glycoprotein spikes on the viral surface, and its head domain is divided into three subdomains (domains I, II, and III, also referred to as domain A, B and β-ribbon) with different arrangements [16,17]. Gc, a class II fusion protein, is oriented away from the viral membrane [14]. Together, these two glycoproteins organize into pentameric or hexameric arrangements of heterodimers on the viral envelope, exhibiting icosahedral symmetry [18,19]. Crystal structure analysis showed that the N-terminal domains of RVFV Gn localize to the membrane-distal region and shields the hydrophobic fusion loops of the Gc, preventing premature fusion [17]. After attachment to the host cells by Gn, virions are internalized through caveolae-mediated endocytosis [20], and transported to the late endosomal compartments [21]. Once exposure to the acidic conditions in the late endosome, Gn repositions, then the exposed fusion loop of Gc interacts with the host membrane initiating the membrane fusion process [17,22,23]. Many studies have shown that neutralizing antibodies (nAbs) against Gn alone or in combination with Gc could provide sufficient protection for animals against RVFV infection, revealing them to be important targets for vaccine and antiviral design [24-27].

Here，we reported the isolation of 77 Gn-specific monoclonal antibodies (mAbs) from rhesus macaques immunized with Ad4-GnGc or Ad5-GnGc by single memory B cells sorting assay. Among these mAbs, 20 exhibited neutralizing activity. These neutralizing antibodies were divided into two groups, with Group A targeting subdomain I and Group B targeting subdomain III of the Gn protein. Representative mAbs A38 and A13 were chosen from these two groups, and both antibodies demonstrated protective efficacy against RVFV challenge in prophylactic and therapeutic mouse models of experimental infection when used as a monotherapy. Research on the neutralization mechanism revealed A13 and A38 block RVFV at different stages of viral infection. Specifically, A13 inhibit membrane fusion, while A38 neutralizes RVFV infection primarily by blocking virus attachment and membrane fusion. Furthermore, we predicted the variable regions of the A38 and A13 and performed molecular docking with RVFV Gn head domain. These structural insights provide evidence that neutralizing antibodies can target distinct stages of the RVFV life cycle. Our findings not only enhance our understanding of viral infection mechanisms but also offer valuable information for the design of novel therapeutics and vaccines against RVFV.

## Results

### Isolation and characterization of neutralizing antibodies for RVFV

**Figure 1.**
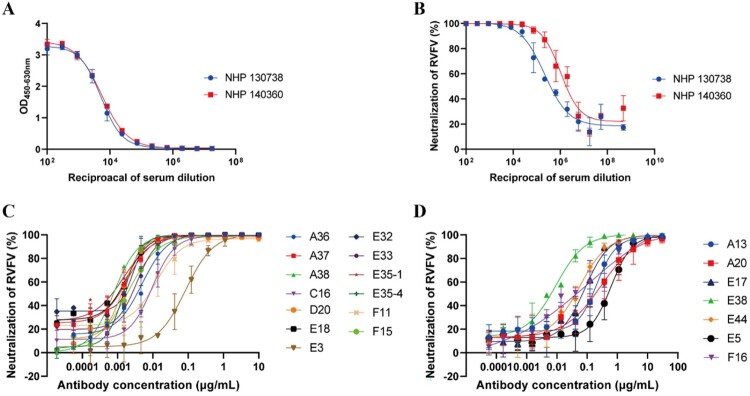
Isolation and neutralizing activity identification of Gn-specific mAbs. Two rhesus macaques (designated NHP130738 and NHP140360) were vaccinated with 10^8^ IFU Ad4-GnGc or 10^8^ IFU Ad5-GnGc respectively on day 0 and boosted with the same dose on day 28. On day 56 and 182, both were vaccinated with 250 μg Gn and 250 μg Gc proteins. PBMCs and serum samples were isolated on day 210 for Gn-specific memory B cells sorting and antibody detection, respectively. (a) RVFV Gn-specific IgG antibody titres were determined by ELISA. (b) Neutralizing antibody titres in sera were measured by a microneutralization assay. (c) and (d) Neutralizing capacities of 20 Gn-specific mAbs. Data are shown as mean ± standard error of mean (SEM) of triplicate wells.

Two rhesus macaques (designated NHP130738 and NHP140360) were vaccinated with either 10^8^ IFU Ad4-GnGc or 10^8^ IFU Ad5-GnGc on day 0 and boosted with the same dose on day 28. On day 56 and 182, both animals received vaccinations with 250 μg of Gn and 250 μg of Gc proteins. Peripheral blood mononuclear cells (PBMCs) and serum samples were collected on day 210 for antibody detection and cell sorting analysis. The results showed that the levels of Gn-specific IgG antibody in both rhesus macaques were comparable (Figure 1(a)), while the neutralizing antibody titre in NHP140360 (IC_50_ 1: 1046158) was higher than that in NHP130738 (IC_50_ 1: 217292) (Figure 1(b)). Subsequently, Gn-specific memory B cells were sorted from PBMCs as single cells using a previously described method (Supplementary Figure 1) [28].

A total of 77 Gn-specific binding antibodies (bAbs) were obtained through single-cell sorting, with 40 derived from NHP130738 and 37 from NHP140360. The neutralization activity of each antibody was determined with the rescued reporter virus rMP-12-eGFP by a microneutralization assay. The results revealed that 4 of 40 bAbs from NHP130738 and 16 of 37 bAbs from NHP140360 exhibited neutralizing activity (Figure 1(c, d) and Table 1). Among these nAbs, 12 exhibited potent neutralizing activity against rMP-12, with IC_50_ values below 10 ng/mL. The analysis of the light chains of these nAbs showed that 7 nAbs belonged to the κ chain, while the remaining 13 nAbs belonged to the λ chain. To further elucidate the interactions between the Gn protein and these nAbs, their binding kinetics were determined using surface plasmon resonance (SPR). And the results indicated that most nAbs presented high binding affinities to Gn protein, with equilibrium dissociation constant (Kd) ranging from 0.37–211.67 nM, except for F11, E3 and E5 (Table 1 and Supplementary Figure 2).

**Table 1.** Characteristics and sources of neutralizing antibodies.

	IC_50_ (ng/mL)	K_d_ (nM)	Group	Type of light chain	Source
F15	1.94	7.55	B1	λ	NHP 130738
F16	103.40	0.37		λ	
F11	12.40	–	B2	κ	
E3	83.40	–		κ	
A38	0.76	0.97	A	λ	NHP 140360
E35-1	1.44	0.88		λ	
D20	1.48	0.90		λ	
E35-4	1.54	0.95		λ	
E18	1.83	3.83		κ	
A37	1.95	10.24		λ	
E32	2.72	1.40		λ	
E33	3.52	8.67		λ	
A36	4.92	211.67		λ	
E38	9.26	24.01		λ	
C16	9.53	129.53	B1	λ	
E44	56.37	2.58		λ	
A20	285.90	10.71		κ	
E17	84.79	53.31	B2	κ	
A13	208.90	0.40		κ	
E5	618.70	–		κ	

### Binding profiles of RVFV nAbs

**Figure 2.**
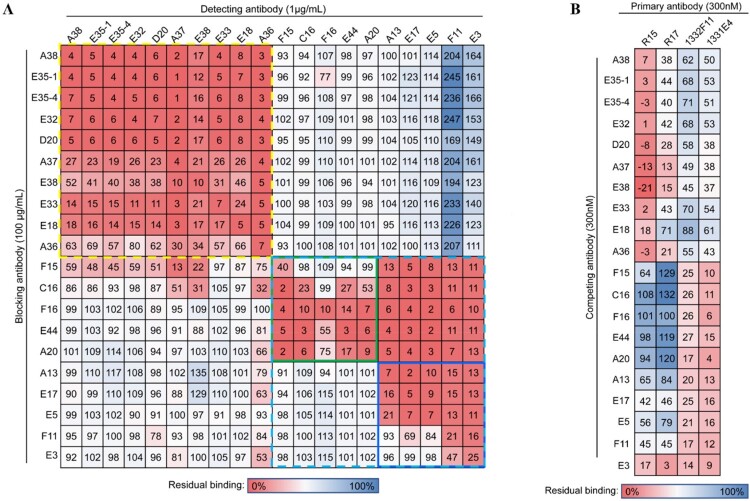
Competition binding of RVFV-specific nAbs. (a) Heatmap showing the competing matrix of 20 nAbs determined by a competition ELISA assay. Numbers in the box indicate the percentage binding of detecting mAb in the presence of the blocking antibody compared with the binding of detecting mAb in the absence of the blocking antibody. Darker red indicates stronger competition, while darker blue indicates weaker competition. The dashed yellow box represents Group A antibodies, and the dashed light blue box represents Group B antibodies. The solid green box represents group B1 antibodies, and the solid blue box represents group B2 antibodies. (b) Heatmap showing the competing matrix between 20 nAbs and representative RVFV antibodies (R15, R17, 1332F11, 1331E4) determined by BLI assay.

To investigate whether these nAbs share overlapping antigenic sites, a competition-binding assay was conducted using ELISA. Two major competition-binding groups were identified, designated Group A (yellow dashed box) and Group B (light blue dashed box). Group B was further divided into two subgroups based on their competitive properties: B1 (green solid box) and B2 (blue solid box) (Figure 2(a)). Group A contains 10 nAbs (A38, E35-1, E35-4, E32, D20, A37, E38, E33, E18 and A36) that exhibit mutual competition, indicating these nAbs recognized similar epitopes (Figure 2(a) and Table 1). Notably, antibodies in subgroup B1 (F15, C16, F16, E44 and A20) can interfere with the binding of subgroup B2 (A13, E17, E5, F11 and E3) to the Gn protein. Conversely, antibodies in subgroup B2 can’t block the binding of subgroup B1 to Gn (Figure 2(a)). Previous structural studies have shown that the extracellular domain of the RVFV Gn protein is composed of a head domain and a stem domain, with the head domain further divided into three subdomains: domain I, domain II, and domain III [16]. To determine which subdomain of the Gn protein is targeted by these nAbs, pre-absorption and binding assays were conducted using Bio-Layer Interferometry, with R15 and R17 reported to target subdomain I, 1332F11 and 1331E4 reported to target subdomain III were used as positive controls [28,29]. Our results showed that group A nAbs exhibit competitive binding activity with R15 and R17, especially with R15, while group B nAbs show competitive binding activity with 1332F11 and 1331E4 (Figure 2(b) and Supplementary Figure 3). These findings suggest that nAbs in Group A target subdomain I of the Gn protein, whereas those in Group B target subdomain III.

**Figure 3.**
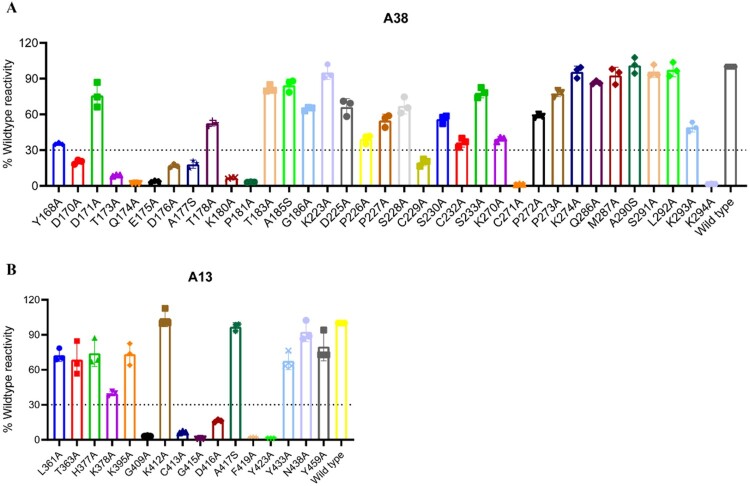
Epitope validation of A38 and A13. The purified Gn protein mutants were coated on a 96-well plate (0.2 μg/well) and incubated with A38(1 μg/mL) or A13(1 μg/mL). Antibody reactivity against each mutant protein was calculated relative to wild-type Gn reactivity by normalizing to the OD_450_/OD_630_ of wild-type. The residues were considered critical if the binding activity relative to wild type < 30%. (a) Binding activity of A38 to Gn protein mutants. (b) Binding activity of A13 to Gn protein mutants.

To identify the key antigenic sites affecting antibody binding, alanine scanning mutagenesis was performed. Based on the competition-binding assay results and the structure of Gn protein, 35 amino acids in domain I and 16 in domain III on the surface of the Gn protein were selected for mutation, with alanine residues mutated to serine. These Gn protein mutants were validated by ELISA using an anti-Gn rabbit polyclonal antibody (Supplementary Figure 4). Next, we assessed the binding capacity of the domain I mutants to A38 and the domain III mutants to A13 using ELISA. The results indicated that residues D170, T173, Q174, E175, D176, A177, K180, P181, C229, C271, and K294 in the N-terminal region of Gn domain I are critical for A38 binding. Additionally, residues G409, C413, G415, D416, F419, and Y423 in domain III are identified as key amino acids for A13 binding (Figure 3). It is worth noting that our choice to mutate four alanine sites (177, 185, and 209 of A38, and 417 of A13) to serine in the two antibodies has certain limitations. The introduction of hydroxyl-containing side chains through these mutations could participate in unintended polar interactions or cause steric clashes, potentially confounding the results. Further experiments, such as mutating alanine to valine at these four sites, are needed to verify the results.

### Genetic characterization of Gn-specific antibody

**Figure 4.**
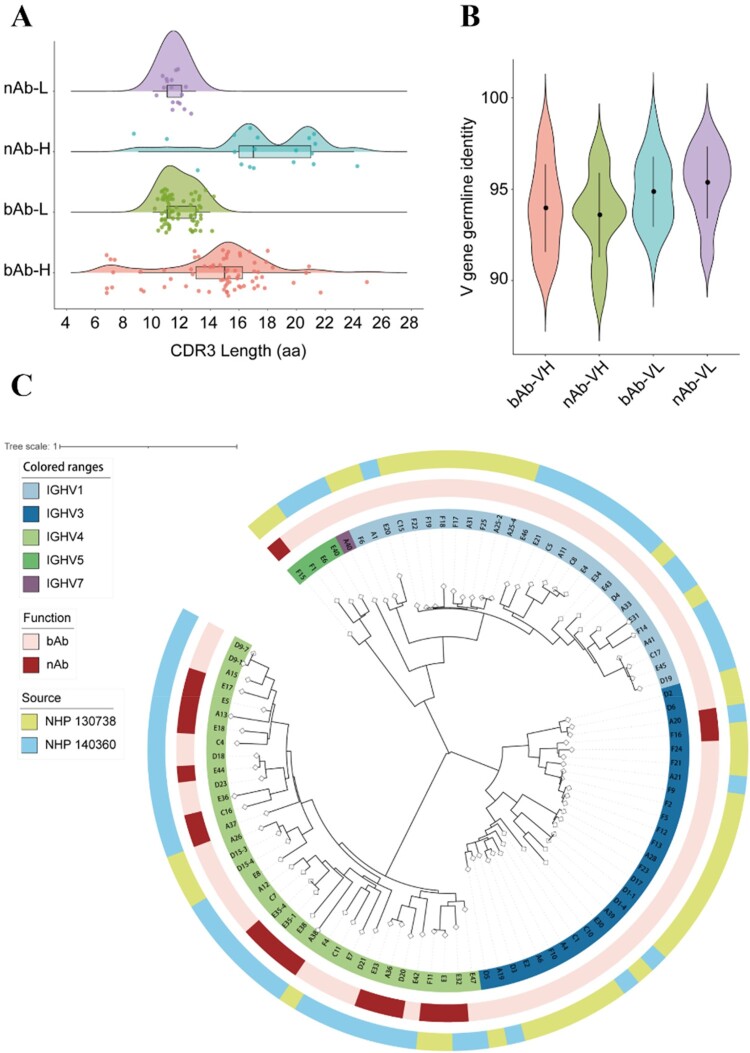
Genetic analysis of Gn-specific antibody. (a) CDR3 amino acid (aa) lengths of VH and VL of nAbs and bAbs. (b) The VH and VL gene identities from the germlines of nAbs and bAbs. (c) The correlation analysis of the phyletic classification, function, and source of Gn-specific antibodies.

To evaluate the genetic characterization of bAbs (without neutralizing activity) and nAbs, the sequences of the variable regions of heavy and light chains (VH and VL) were analyzed. The results showed that the median length of CDR3 region in VH of nAbs is significant longer than that of bAbs (*P* = 0.0008), while the length of CDR3 region in VL of both types of antibodies are similar (*P* = 0.33) (Figure 4(a)), indicating a potential correlation between the length of the CDR3 region in VH and the neutralizing activity of antibodies. The VH gene germline identity of bAbs (average of 93.97%) is higher than that of nAbs (average of 93.59%), whereas nAbs exhibit higher VL gene germline identities (average of 95.37%) compared to bAbs (average of 94.86%) (Figure 4(b)). To further investigate the evolutionary relationship between the bAbs and nAbs, the sequences of VH and VL of bAbs and nAbs were analyzed. And the result of heavy chain clonotypes analysis revealed that the VH of bAbs and nAbs were distributed in five IGHV gene families, with the VH of bAbs mainly distributed in IGHV1, IGHV3 and IGHV4, and the VH of nAbs mainly distributed in IGHV4 (Figure 4(c)). According to the pairing information of VH and VL gene family, the light chains of bAbs principally come from IGKV1, IGKV2, IGLV1 and IGKV3 (Supplementary Figure 5A), while the light chain of nAbs mainly originate from IGKV1, IGKV3, IGLV11 and IGLV5 (Supplementary Figure 5B).

It's worth noting that a significant difference in the quantity of nAbs produced by two rhesus macaques was observed, although the vaccines they received were both able to induce strong cellular and humoral immune responses [26,30]. We speculated that this phenomenon might arise from two potential causes: first, the individual differences in immune responses between macaques; second, the varying reactivity of the host to different antigen carriers (Ad4-GnGc and Ad5-GcGn). Our studies have shown that the VH of the RVFV nAbs mainly originated from IGHV4. Given the high variability of the gene and allele of antigen receptor sites among different individuals, this may affect the quality of the immune response to different pathogens. Moreover, the usage of IGH alleles can influence the activity of nAbs [31,32]. Therefore, we hypothesize that IGHV4 may play a key role in the formation of RVFV nAbs.

### Prophylactic and therapeutic effects of nAbs in vivo

**Figure 5.**
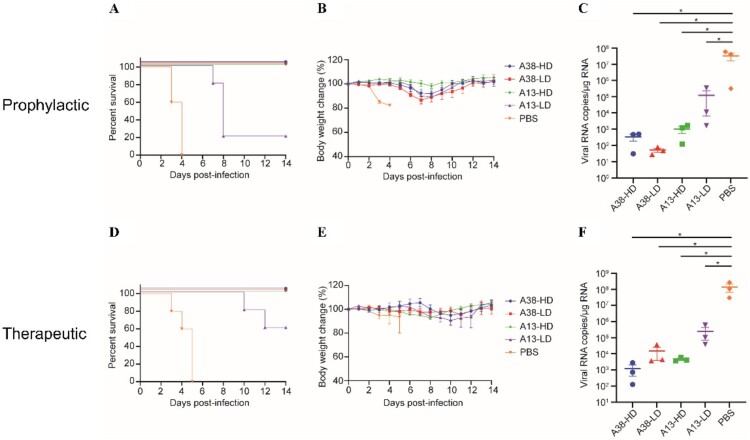
Protection potency of A38 and A13 when used as prophylaxis or therapy against lethal RVFV infection in A129 mice. For prophylactic treatment, mAbs (200 μg or 20 μg) or PBS was administered once by the intraperitoneal route to A129 mice (n = 8) 24 h prior to subcutaneous inoculation with 2 × 10^4^ TCID_50_ of RVFV rMP-12 strain. Two days after challenge, three mice from each group were euthanatized and their livers were collected for viral RNA load detection. (a) Survival rate of mice (n = 5). (b) Body weight changes after challenge (n = 5). (c) Viral loads in the livers two days after challenge were determined by qRT–PCR (n = 3). For therapeutic treatment, mice (n = 8) were challenged subcutaneously with 2 × 10^4^ TCID_50_ of RVFV rMP-12 strain 24 h before intraperitoneal administration of mAbs (200 μg or 20 μg) or PBS. Three days after challenge, three mice from each group were euthanatized and their livers were collected for viral RNA loads determination. (d) Survival rate of mice (n = 5). (e) Body weight changes after challenge (n = 5). (f) Viral loads in the livers three days after challenge were determined by qRT-PCR (n = 3). Data are shown as the mean ± SEM. *P* values were calculated by one-way ANOVA with multiple comparison tests. *P < 0.05.

Based on the criteria of strong competitive binding activity within the group, high affinity for the Gn protein, and potent neutralization activity, monoclonal antibodies A38 and A13 were selected for further research. To evaluate the prophylactic and therapeutic effects of these two neutralizing antibodies, interferon-α/β receptor-deficient A129 mice were chosen as the animal model. These mice are highly permissive to lethal RVFV challenge and are widely used to assess the protective efficacy of vaccines [25,33]. To evaluate the prophylactic effects of the two antibodies, groups of mice (n = 8) were administered intraperitoneally with 200 μg (high dose, HD) or 20 μg (low dose, LD) of A38 or A13. Twenty-four hours post-administration, the mice were challenged subcutaneously with 2 × 10^4^ TCID_50_ of RVFV rMP-12 strain. Three mice from each group were euthanized 2 days post-infection for viral loads determination in the liver. The remaining mice were monitored for morbidity and mortality over a 14-day period following infection. The results showed that all mice in the PBS control group succumbed to infection within 4 days. In contrast, all mice treated with the antibodies survived, except for the A13-LD group, where only one mouse survived (Figure 5(a)). Compared with the PBS group, all survived mice displayed similar body weight change, with a slight weight loss at 4–7 days after infection, followed by a rapid increase to normal levels (Figure 5(b)). Additionally, viral loads in the antibody-administered groups were significantly reduced compared to those in the PBS group (Figure 5(c)).

To evaluate the effects of A38 and A13 when used as postexposure therapies, groups of mice (n = 8) were inoculated subcutaneously with RVFV. Twenty-four hours post-challenge, the mice were administered intraperitoneally with 200 μg (HD) or 20 μg (LD) of A38 or A13. Three mice from each group were euthanized at 3 days post-infection for viral loads determination in the liver. As expected, the therapeutic administration of these antibodies significantly improved survival rates and weight maintenance, with mice in the A38-HD, A38-LD and A13-HD therapeutic groups all survived the infection, although two mice in A13-LD group succumbed, the time to death was significantly prolonged compared to the PBS group (Figure 5(d)). Besides, the survived mice exhibited minimal weight fluctuations and the viral loads in livers were significantly decreased when compared with the PBS group (Figure 5(e, f)).

### A38 and A13 block virus infection by targeting different stages of the viral life cycle

**Figure 6.**
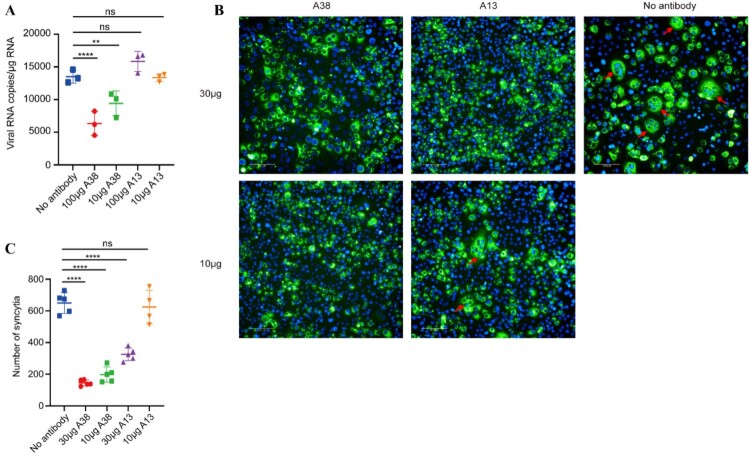
Neutralizing mechanism of A38 and A13. (a) The ability of A38 and A13 to block RVFV rMP-12 attach to Vero E6 cells. Vero-E6 cells were treated with 50 nM Bafilomycin A1 at 37 °C for 1 h. At the same time, 2 × 10^4^ TCID_50_ rMP-12 was co-incubated with 100 μg or 10 μg A38 or A13 antibody at 37 °C for 1 h. Then the virus-nAbs mixtures were added to the cells and incubated at 37 °C for 1 h. After rinse, the cells were analyzed for viral RNA loads by qRT-PCR. (b) and (c) The ability of A38 and A13 to inhibit membrane fusion. Vero E6 cells were infected with Ad5-GnGc at MOI of 10 for 12 h, then the infected and uninfected Vero E6 cells were co-cultured. After that, 10 μg or 30 μg A38 or A13 antibody was added to the cells and cultured at 37 °C for 1 h. After that cells were treated with mildly acidic PBS (pH 5.3) at 37 °C for 20 min and cultured at 37 °C for 5 h. Then Cells were fixed, Gn protein was staining with specific monoclonal antibodies (Green), and nuclei were stained with DAPI (blue). The syncytia were observed (b) and counted (c) by High Content Analysis System. The red arrow represents the syncytium. Data are displayed as the mean ± SEM. *P* values were calculated by one-way ANOVA with multiple comparison tests. **P < 0.01 ****P < 0.0001.

Previous research has elucidated that R15 and R17 antibody mediated neutralization by blocking RVFV adsorption to host cells [29]. Considering that A38 exhibits competitive binding activity with R15 and R17 (Figure 2(b) and Supplementary Figure 3), we hypothesized that A38 might exert neutralization through the same mechanism. To test this hypothesis, the attachment inhibition assay was performed according to the previously reported procedure [20,22]. In this assay, Vero E6 were pre-treated with 50 nM Bafilomycin A1 at 37 °C for 1 h to block viral-cell membrane fusion without interfering with viral adsorption to the cell surface. Our results showed that A38 significantly reduced the number of viruses attached to cells, while A13 had no effect at all, indicating that A38 exerts neutralization by inhibiting adsorption, while A13 likely employs a distinct mechanism (Figure 6(a)).

Nathaniel et.al have reported that several RVFV antibodies mediate neutralization through membrane fusion inhibition [34]. Given that some of the epitopes recognized by these neutralizing antibodies are in close proximity to those recognized by A38 and A13, we sought to comprehensively investigate the neutralization mechanisms of these two antibodies. To this end, we developed an RVFV cell–cell fusion assay based on previous research, which demonstrated that treatment of RVFV GnGc-expressing cells with mildly acidic media (pH 6.2 or below) could induce rapid and efficient syncytia formation [35]. To verify this phenomenon, Vero E6 cells infected with Ad5-GnGc (MOI = 10) to express RVFV GnGc protein served as effector cells, while untreated Vero E6 cells were used as the target cells. After co-incubation in 96-well plate for 24 h, cells were treated with mildly acidic PBS (pH 5.3) for 20 min, then incubated at 37 °C for 5 h. The results showed that acid treatment (pH 5.3) induced extensive syncytia formation, whereas no syncytia were observed under neutral conditions or in the Ad5-empty control group (Supplementary Figure 6).

Considering that A38 and A13 may need to function in this acidic environment, we first tested their binding capacity to the Gn protein in this condition by ELISA. And the results indicated that A38 and A13 could still maintain good binding activity with Gn protein in this challenging environment (Supplementary Figure 7). To further explore whether these antibodies could interfere with syncytia formation, A38 and A13 were incubated with effector-target cells for 1 h following co-culture of effector and target cells. After treated with acidic PBS and incubated for 5 h, indirect immunofluorescence assay (IFA) was performed, and the number of syncytia was counted. The results showed that except for the low-dose group of A13 with sporadic syncytia observed, no syncytia could be found in the other antibody treated groups, indicating that A38 and A13 could exert neutralization through fusion inhibition (Figure 6(b, c)). Collectively, our data indicate that A38 mediates neutralization through dual mechanisms of blocking viral attachment and inhibiting membrane fusion, whereas A13 primarily exerts its neutralizing effect via fusion inhibition. It is worth noting that previous research has shown that bivalent recognition is essential for effective fusion inhibition [34], whether the same phenomenon exists in A38 and A13 deserves further investigation.

### Molecular determinants of A38 and A13

**Figure 7.**
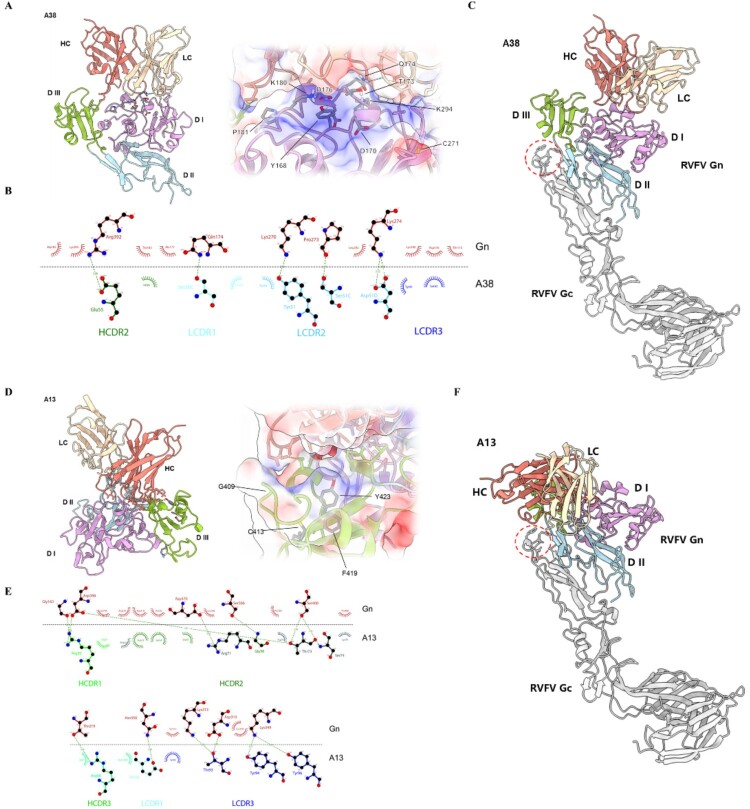
Structures of Gn head domain with variable regions of A38 and A13. The variable regions of antibodies were predicted by ABodyBuilder2, followed by molecular docking with protein structure of the RVFV Gn head domain (PDB ID: 5Y0W) via the ClusPro web server. (a, d) Interactions between variable regions of A38 and A13 with the RVFV Gn. Left, The Gn head is presented as a cartoon diagram and subdomains I, II and III were coloured violet, sky blue and green, respectively. HC and LC were shown as cartoon and coloured red and pale yellow, respectively; Right, zoom-in view of binding interface, The dashed lines indicate hydrogen bond. Surface colouring (blue/red) indicates the charge distribution at the interaction interface. (b, e) 2D diagram of variable regions of antibodies paratope and epitope residues involved in hydrogen bonds (green dashed lines, distances in Å). Atoms are shown as circles, with oxygen, carbon and nitrogen in red, black and blue, respectively. Interacting residues that belong to CDR loops are marked below. (c, f) Superposition of Gn/A38 and Gn/A13 onto the RVFV Gn/Gc complex. Gn and Gc domains were shown as cartoons, with Gc coloured as grey. The Gc fusion loop is circled with red dashed lines. The colouring of Gn is consistent with the colours in A and D.

To clarify the molecular basis for the neutralizing activity of A38 and A13 against RVFV, the variable regions of antibodies were predicted by ABodyBuilder2 [36], followed by molecular docking with protein structure of the RVFV Gn head domain (PDB ID: 5Y0W) via the ClusPro web server [37]. The structural models of the antigen–antibody complexes depicted in Figure 7 were selected as the optimal conformation based on the ranking of docking poses from ClusPro output and validated by the results of alanine scanning mutagenesis and competition-binding assays. Based on this hypothetical model, we conducted in-depth research on the interaction pattern between A13/A38 and the Gn protein, as well as the corresponding neutralization mechanism. The RVFV Gn head domain is composed of three subdomains, DI, DII and DIII. Competition-binding assays confirmed that A38 and A13 interact with these subdomains through distinct mechanisms (Figure 2), with minimal overlap in their binding motifs. A38 Fab binds exclusively to the Gn DI and DIII subdomains on the RVFV surface (Figure 7(a)), whereas A13 Fab recognizes an epitope spanning all three subdomains at the interface between adjacent RVFV protomers (Figure 7(d)).

Analysis of the antigen–antibody interaction interface revealed that A38 engages in extensive charge and hydrogen bond interactions with Gn. Specifically, hydrogen bonds form between RVFV Gn residues (Gln174, Lys270, Pro273, Lys274, Arg329) and A38 light chain residues (Ser30C, Tyr51, Ser51C, Asp51D), along with Glu55 of the A38 heavy chain (Figure 7(a, b)). Also, an extensive hydrogen bond network is formed between specific residues of RVFV Gn (Gly163, Thr219, Ser306, Asp310, Lys313, Lys349, Asn350, Asp370, Asp398, Ser400) and residues from A13, including Arg30, Gly56, Arg71, Thr73, Ser74 and Arg98 from the HC, as well as Glu32, Thr93, Tyr94 and Tyr96 from the LC (Figure 7(d, e)). The greater number of hydrogen bonds formed between A13 and Gn might correlate with its higher binding affinity compared to A38. Besides, our analysis revealed that the binding interfaces for both A38 (HC/LC) and A13 (HC) are characterized by a hydrophobic microenvironment (Supplementary Figure 8). The binding affinity between the antibodies and Gn is also influenced by hydrophobic interactions, particularly involving the hydrophobic residues enriched in the antibody's CDR3 region. These findings provide molecular-level confirmation and refinement of the antibody binding characteristics described above.

Functional studies demonstrated that A13 neutralizes RVFV by inhibiting viral fusion (Figure 6). On the virion surface, Gc interacts with Gn subdomain II and lines the inner half of the glycoprotein shell beneath Gn. Under neutral pH conditions, the Gc fusion loop is shielded from solvents by Gn DI and DII [17]. Superposition of the predicted RVFV Gn/A13 complex and the known RVFV Gn/Gc complex structure (PDB ID: 6F9C) shows that A13 engages in extensive interactions with Gn subdomains and creates steric hindrance for the rearrangement process, which blocks the exposure of the Gc fusion loop, thereby inhibiting Gc-mediated membrane fusion (Figure 7(f)). Cell-based assays demonstrated that A38 neutralizes RVFV by inhibiting both viral attachment and membrane fusion (Figure 6). Since A38 interacts with Gn on the virion surface and its binding footprint may overlap with the cell receptor binding motif, it is evident how A38 inhibits viral attachment. Based on superposition of Gn/A38 complex and the known RVFV Gn/Gc complex structure, the A38-epitope binding area is positioned above the Gc fusion loop (Figure 7(c)). In the endosomal environment, low pH triggers conformational changes in viral surface proteins, leading to the exposure of the hydrophobic Gc fusion loop and its insertion into the host cell membrane [23]. The binding of A38 to Gn likely stabilizes the overall structure of the RVFV glycoprotein in the low pH environment, thereby preventing the exposure of the Gc fusion loop and mediating viral neutralization.

It is important to note that the above analysis of the A38/A13 – Gn interaction interface and neutralization mechanism is based on a single, optimized docking conformation derived from multiple data sources. While this model provides a key framework for us to elucidate the neutralization mechanism of A13 and A38 at the molecular level, it remains a postulate that requires experimental validation. Consequently, all specific mechanistic interpretations derived from it need to be verified through further studies.

## Discussion

The glycoproteins Gn and Gc on the surface of RVFV are the primary targets of nAb responses during natural infection and vaccination, as demonstrated by our and many other researches [25,29,38,39]. Our prior research showed that human adenovirus serotypes 4 and 5 expressing codon-optimized RVFV glycoproteins Gn and Gc could induce robust immune responses in mice [26,30]. In this study, we extended these findings by vaccinating rhesus macaques with these adenovirus-based vaccines. The results demonstrated that these vaccines also elicited strong antibody responses in non-human primates, further validating their potential as effective immunization strategies against RVFV. We characterized memory B cells encoding monoclonal antibodies targeting RVFV Gn and Gc proteins. Among the twenty Gn-specific nAbs, seven incorporated κ light chains, thirteen incorporated λ light chains. We suspected this distribution may be attributed to the different efficiencies of primers used in PCR amplification. Similar λ light chain-biased antibody responses were also reported in humans infected with RVFV, as well as in ferrets and humans infected with influenza virus [29,40,41]. Further research is needed to explain this phenomenon and elucidate the underlying mechanisms.

The twenty nAbs identified were divided into two groups (Group A and Group B) based on their competition-binding characteristics. Group B was further subdivided into two subgroups (B1 and B2) based on distinct competitive interactions. Notably, antibodies in subgroup B1 can interfere with the binding of subgroup B2 to Gn, while antibodies in subgroup B2 can’t block the binding of subgroup B1 to Gn. We speculate that this phenomenon may arise from differences in the affinity of antibodies for Gn, with subgroup B2 antibodies exhibiting lower affinity compared to those in subgroup B1. The crystal structure of the Gn head has been analyzed by different studies and named differently. Yan et.al have determined the crystal structure of the Gn head and divided it into three subdomains: domains I (residues154-300), II (residues 301–365 and 442-470), and III (residues 366-441) [16]. Steinar Halldorsson et.al named the three domains of Gn head: domain A (residues 154–305), a β-ribbon domain (residues 306–365 and 440–469) and a small globular domain B (residues 366–439) [17]. In this study, the first name was used. Four mAbs R15, R17, 1332F11 and 1331E4 with known epitopes were used as positive controls. Our results showed that group A have competitive binding activity with R15 and R17, while group B have competitive binding activity with 1332F11 and 1331E4. Two nAbs (A13 and A38) were chosen from each group and the protection efficacies of these two antibodies against RVFV infection were evaluated in a mouse model. The results showed that representative nAbs A38 and A13 exhibited protection efficacies against RVFV infection in both prophylactic and therapeutic settings, with A13 exhibiting slightly weaker protection ability at low dose. Notably, the A38 groups did not exhibit a clear dose–response relationship. We attribute this to the complete protection achieved even at the low dose, which resulted in no significant differences in measured parameters between the HD and LD groups (Figure 5). Although the viral load in the HD group was slightly higher (Figure 5(c)), this difference was not statistically significant. This likely occurred because viral levels in both groups were effectively reduced to extremely low concentrations near the assay's limit of quantification, making the observed variation more likely attributable to technical variability rather than biologically relevant differences. Besides, in the A13-LD group, neither prophylactic nor therapeutic administration achieved complete protection. Although a higher survival rate was observed in the therapeutic group, suggesting a potential trend toward better efficacy, this difference was not statistically significant. Given that the current study included only two dose groups, the relative efficacy of different administration strategies remains inconclusive. Elucidating the efficacy of antibodies under different administration routes is a valuable research direction. Therefore, future studies should incorporate additional dose groups with larger sample sizes to systematically determine the minimum effective dose for complete protection and to clarify the efficacy difference between prophylactic and therapeutic use of A13.

Neutralizing antibodies work by blocking different stages of the viral life cycle, with blocking adsorption and inhibiting membrane fusion being the two most common ways of function. To investigate the neutralization mechanism of A13 and A38, we first assessed whether these two antibodies could block virus adsorption, a mechanism commonly employed by most neutralizing antibodies against RVFV and other viruses [29,42,43]. And the results of attachment inhibition assay indicated that A38 exerts neutralization by inhibiting adsorption, while A13 can’t block virus attachment. Next, to explore whether these two nAbs could inhibit membrane fusion, we developed an RVFV cell–cell fusion assay based on the previous research [35]. RVFV glycoproteins are targeted to the Golgi apparatus and are not efficiently delivered to the cell surface. However, studies have shown that overexpression of RVFV glycoproteins using an alphavirus replicon vector could resulted in the expression of the glycoproteins on the surface [35]. A similar phenomenon was observed when Vero E6 cells were infected with Ad5-GnGc. Normally, the fusion of viruses and cells occurs in the acidic environment of endosomes [17]. However, researchers have demonstrated that exposing viruses to low pH at the plasma membrane surface could induce fusion of RVFV [34]. Besides, treating cells expressing RVFV glycoproteins with mildly acidic media could lead to rapid and efficient syncytia formation [35]. Given the above results and the difficulty in directly observing endosomal membrane fusion, we utilized Ad5-GnGc-infected Vero E6 cells as effector cells. These cells were co-cultured with untreated Vero E6 cells and subjected to acid treatment. The experiment resulted in extensive syncytium formation, which could be quantitatively assessed. This approach effectively visualized the membrane fusion process that typically occurs within endosomes, minimizing the need for handling high-risk viruses. It enables high-throughput studies of RVFV glycoprotein fusion activity and facilitates the screening of small molecule inhibitors that block virus-cell fusion. Using this method, we confirmed that A38 and A13 can inhibit RVFV fusion to cells. However, one limitation of this study is that we did not extensively investigate the neutralization mechanisms of other antibodies in the two groups beyond A38 and A13. Although the competition ELISA and BLI competition assay results suggest that the binding epitopes of A38 and A13 with other antibodies within their respective groups are quite similar, and thus their neutralization mechanisms might also be similar, this remains speculative. The precise mechanisms require further experimental validation.

To dissect the mechanisms of these two neutralizing antibodies at the molecular level, we predicted the variable regions of antibodies and performed molecular docking with RVFV Gn head domain. Structural analysis reveals that, in addition to Gn subdomain I, A38 also interacts with the DIII subdomain. This interaction may stabilize the overall glycoprotein structure and impede the exposure of the Gc fusion loop during viral infection (Figure 7(a, c)). In contrast, A13 exerts its membrane – fusion inhibitory effect by forming extensive interactions with Gn and directly contacting the Gc fusion loop. (Figure 7(d, f)). Neutralizing antibodies that can block viral infection at multiple stages of the virus life cycle have been identified for several viruses. For example, antibodies such as C9 and IM-CKV063 have been shown to simultaneously inhibit both the entry and release of Chikungunya virus (CHIKV) [44]. Similarly, CHE19 has been demonstrated to suppress viral fusion and release of CHIKV [45]. Additionally, antibodies like S2A5, S1G3, and S1H7 have been found to block multiple steps during Severe Fever with Thrombocytopenia Syndrome Virus (SFTSV) infection, including viral attachment and membrane fusion [46]. Previous studies have revealed that RVFV Gn-specific monoclonal antibodies can prevent infection by blocking virion attachment to host cells or by inhibiting membrane fusion [29,34,47]. However, to date, no studies have reported neutralizing antibodies that target multiple steps in the RVFV life cycle. Therefore, our study is the first to demonstrate that the screened nAb can inhibit RVFV infection at multiple stages of its life cycle. It seems that the results of the structural analysis are somewhat divergent from the above competition-binding assay results. However, it should be noted that the main binding regions of nAbs to the Gn protein were primarily deduced from competitive experiments using literature – reported antibodies, which inevitably imposes certain limitations on these findings. Despite this, the structural analysis has, to a certain extent, confirmed and refined the aforementioned results. Overall, our findings not only offer a more comprehensive understanding of the neutralizing mechanisms of RVFV antibodies but also provide valuable insights to guide the development of vaccines and therapeutics against RVFV infection.

## Materials and methods

### Cells, viruses, and animals

Vero E6 cells (African green monkey kidney, ATCC) were cultured in Dulbecco’s modified Eagle’s medium (DMEM) (Gibco, Grand Island, NY, USA) supplemented with 10% fetal bovine serum (FBS) (Gibco, Grand Island, NY, USA) and maintained at 37°C with 5% CO_2_. Ad4-GnGc [26] and Ad5-GnGc [30] were constructed and preserved at Beijing Institute of Biotechnology. RVFV MP-12 strain and RVFV MP-12 strain expressing eGFP were rescued utilizing a previously described method [48] and named rMP-12 and rMP-12-eGFP, respectively.

The interferon-α/β receptor-deficient A129 mice were preserved and housed in the animal facility of the Animal Center, Beijing Institute of Biotechnology. RVFV challenge experiments were performed in animal biosafety level 2 (ABSL-2) facilities at Beijing Institute of Biotechnology. Two healthy rhesus macaques (females, between 5–6 years old) were purchased and housed at the Beijing Institute of Xieerxin Biology Resource.

All animal experiments were approved by the Animal Care and Use Committee of Beijing Institute of Biotechnology, China (permit number for mouse experiments: IACUC-SWGCYJS-2021-006, permit number for rhesus macaque experiments: E20181030) and conducted in strict accordance with the Guide for the Care and Use of Laboratory Animals of the People’s Republic of China.

### Protein expression and purification

The codon optimized gene of Gn protein (M segment 154–469aa, GenBank accession number: DQ380208.1) was cloned into pCAGGs plasmid, with a tissue plasminogen activator (tPA) signal peptide at the N-terminal and a Strep-tag II at the C-terminal. After transfection into Expi293F cells for four days, the cells were harvested and lysed via ultrasonic waves. Then, the Gn protein was purified by affinity chromatography with a 5-mL StrepTrapTM HP column (GE Healthcare, Chicago, IL, USA). The DNA encoding the Fab fragment of A38 or A13 was cloned into the pcDNA3.1 vector (Novagen) and transfected into the Expi293F mammalian cells for protein expression. Subsequently, the protein was purified by Nickle column and gel-filtration on a HiLoad 16/600 Superdex 75 pg column (Cytiva).

### Enzyme-linked immunosorbent assay (ELISA)

The Gn protein (0.2 μg/well) was coated on 96-well microplates at 4 °C overnight. The plates were rinsed three times with PBST (PBS containing 0.2% tween 20) and blocked with 2% BSA at 37 °C for 1 h. After washing with PBST, the three-fold serial dilutions of monkey serum samples (starting at 1:100 dilution) or purified monoclonal antibodies (starting at 10 μg/mL) in PBST were added to the microplates and incubated for 1 h at 37 °C. Washed again, for monkey serum samples, anti-monkey IgG antibody conjugated to horseradish peroxidase (HRP) purchased from Abcam (ab112767, 1:10000) was added to the microplates and incubated at 37 °C for 1 h. For purified monoclonal antibodies, anti-human IgG antibody (HRP) purchased from Abcam (ab97225, 1:10000) was added to the microplates and incubated for 1 h at 37 °C. Subsequently, the plates were washed with PBST, the TMB substrate was added to the microplates and incubated 5 min at room temperature, and finally 2 M H_2_SO_4_ was added to stop the reaction. The optical density (OD) was determined at 450 nm/630 nm using a microplate reader (SPECTRA MAX 190, Molecular Device).

### Neutralization assay

The neutralizing activity of the monkey serums or monoclonal antibodies against RVFV were measured through a microneutralization assay as previously described [26]. The serum samples were heated at 56 °C for 30 min and serially diluted threefold starting from 1:100. The monoclonal antibodies were serially diluted threefold starting from 10 μg/mL or 30 μg/mL. The serial dilution sera or monoclonal antibodies were incubated with equal volume 100 TCID_50_ of rMP-12-eGFP in 96-well plates at 37 °C for 1 h. After that, 20,000 Vero E6 cells were added to each well and incubated at 37 °C with 5% CO_2_ for 48 h. Then, the cells were fixed with 4% paraformaldehyde at room temperature for 3 h. Afterward, the cells were rinsed three times with PBS and stained with DAPI (Thermo Fisher Scientific, 1:1000). The quantities of infected cells (eGFP) and total cells (DAPI) were measured through the Celigo imaging cytometer (Nexcelom, Boston, MA, USA). The neutralizing antibody titres of serum samples and the half maximal inhibitory concentration (IC_50_) values of monoclonal antibodies were calculated via a sigmoidal, four parameter logistic nonlinear fit analysis in software GraphPad Prism 8.

### Gn-specific B cell sorting

Gn-specific single memory B cells were isolated according to a method previously described [28]. Briefly, PBMCs were stained with anti-human CD3-PerCP (BD, 552851, 1:250), anti-human CD19-Alexa Flour 700 (BD, 557921, 1:200), anti-Human IgG-PE (BD, 555787, 1:500), and Gn protein containing Strep-tag (2 μg/5×10^5^ cells) at 4 °C for 1 h. After washing twice with PBS containing 2% FBS, the PBMCs were stained with APC Streptavidin (Biolegend, 405243, 1:500) at 4 °C for 1 h. Gn-specific B cells were defined as CD19^+^ CD3 ^–^ hIgG^+^ Strep^+^ and sorted into 96-well plates (one cell per well) using a MoFlo XDP cell sorter (Beckman Coulter).

### Gn-specific antibodies sequencing data analysis

The V gene segments of the antibody sequences were annotated with standalone IgBLAST1.15.0 [49] and a sequence database of germline gene segments from the international ImMunoGeneTics information system (IMGT) [50]. The CDR3 region amino acid sequence length of antibodies can be further visualized in R using ggplot2 version 3.3.3 and ggridges version 0.5.3 packages. Mutation frequencies in V genes were calculated using the calcObservedMutations function from Immcantation/SHazaM v1.0.2 R package. Violin graphics were obtained using the ggpubr version 0.4.0 packages. Combination of V gene family of heavy chain and light chain V gene family pairing information of antibodies was processed using R and graphic was obtained using the circlize v0.4.12 packages in R v3.6.3. Multiple Sequence Alignment by CLUSTALW (https://www.genome.jp/toolsbin/CLUSTALW) web is applied as our sequence alignment tool. Amino acid sequences of HCDR3 and LCDR3 in neutralizing and binding antibodies were submitted to CLUSTALW in PROTEIN and SLOW/ACCURATE mode. The result of the multiple sequence alignment was provided to GenomeNet (https://www.genome.jp/toolsbin/ete). Phylogenetic analysis pipeline performs by ETE3 v3.1.1, in which, ML tree was inferred using PhyML v20160115. And the evolutionary tree was refined and visualized using the iTOL (interactive Tree of Life) online tool (https://itol.embl.de/).

### Surface plasmon resonance (SPR) analysis

The binding kinetics between nAbs and Gn protein were determined by SPR utilizing a Biacore T200 device (Cytiva). These nAbs were diluted with HBS-EP + (Cytiva) to a concentration of 0.5 μg/mL and loaded to Protein A chip (Cytiva) at flow rate of 10 μL/min for 60 s. Then the Gn protein was serially two-fold diluted from 1280 nM to 5 nM wtih HBS-EP+, combined with nAbs on chip at flow rate of 10 μL/min for 120 s and dissociated for 600 s at same flow rate. The results were generated through 1:1 binding model fitting.

### Competition ELISA

nAbs were labelled with HRP (Abcam) according to the manufacturer’s protocol. 96-well microplates were coated with Gn protein (0.2 μg/well) at 4 °C overnight. Following rinsing with PBST for three times, the plates were blocked with 2% BSA at 37 °C for 1 h and washed again. After that, the blocking antibodies (unlabelled nAbs) were added to the microplates at a concentration of 100 μg/mL and incubated at 37 °C for 1 h. The unbound blocking antibodies were discarded, and the detecting antibodies were added to the microplates at a concentration of 100 μg/mL and incubated at 37 °C for 1 h. After washing with PBST, the TMB substrate was added to the microplates and incubated 5 min at room temperature, and finally 2 M H_2_SO_4_ was added to stop the reaction. The optical density was determined at 450 nm/630 nm. Results were exhibited as the percent of OD in the presence of blocking nAbs over the OD in the detecting nAbs-only (maximal binding).

### Biolayer interferometry (BLI) competition assay

The BLI competition assay was performed using a Gator® Prime device (Gator Bio). Gn protein was labelled using EZ-Link Sulfo-NHS-LC-LC-Biotin (Thermo Fisher Scientific) according to the manufacturer’s protocol. The biotinylated Gn protein was diluted to 100 nM with PBS containing 0.02% Tween-20 and 1 mg/mL BSA, and loaded onto the streptavidin biosensors (Gator Bio) for 120 s at 400 rpm prior to baseline equilibration for 120 s. Association of primary antibodies (R15, R17, 1332F11 and 1331E4) at 300 nM was performed for 120 s prior to association of competing antibodies at 300 nM for 240 s.

### In vivo animal challenge experiment

The RVFV challenge experiment of interferon-α/β receptor-deficient A129 mice, aged 6–8 weeks, was conducted in BSL-2 facility. In the prophylactic experiment, A129 mice (n = 8, five for clinical signs observation, three for viral loads detection) were administrated intraperitoneally with either 200 μg or 20 μg A38 or A13 antibody 24 h before challenged subcutaneously with 2 × 10^4^ TCID_50_ rMP-12. In the therapeutic experiment (n = 8), 24 h after subcutaneously challenged with 2 × 10^4^ TCID_50_ rMP-12, A129 mice were intraperitoneally administrated with either 200 μg or 20 μg A38 or A13 antibody. The mice weights and survival (n = 5) were monitored daily for 14 days post-infection. In addition, mouse liver tissues both in prophylactic group (n = 3) and in therapeutic group (n = 3) were collected for viral loads detection at 2 and 3 days after infection, respectively.

### Quantitative RT-PCR

The viral loads in the liver of A129 mice were measured by qRT-PCR. Total RNA was extracted via a RNeasy Mini Kit (Qiagen) and reverse transcribed to cDNA by PrimeScript^TM^ RT Master Mix (Takara). Then, the viral copies targeting the L fragment of RVFV were determined by qRT-PCR using TaqMan™ Universal Master Mix II (Thermo Fisher Scientific) with the following probe and primers [51]: forward primer 5′-GAAAATTCCTGAAACACATGG-3′, reverse primer 5′-ACTTCCTTGCATCATCTGATG-3′, and probe 5′-FAM-CAATGTAAGGGGCCTGTGTGGACTTGTG-BHQ1-3′.

### Mutagenesis epitope mapping

As previously described for the construction of RVFV Gn mutation library [34], 51 Gn protein mutants were generated based on RVFV MP-12 fused with a C-terminal Strep-tag II. The expression plasmids of mutants were transfected into Expi293F cells. The cells were harvested four days after transfection and lysed via ultrasonic waves. Then, the Gn protein mutants were purified via StrepTrap™ HP column (GE Healthcare, Chicago, IL, USA). These mutants (0.2 μg/well) were coated on 96-well microplates at 4 °C overnight and rinsed three times with PBST (PBS containing 0.2% tween 20). After blocked with 2% BSA at 37 °C for 1 h, the plates were washed again. Then A38 and A13 were diluted to 1 μg/mL, added to the microplates, and incubated at 37 °C for 1 h. Following that, the plates were washed with PBST and the anti-human IgG antibody (HRP) was added to the microplates and incubated at 37 °C for 1 h. Again, the plates were washed, and TMB substrate solution was added. The reactions were stopped by 2 M sulphuric acid. The OD was determined at 450 nm/630 nm. Antibody reactivity against each mutant segment was calculated relative to wild-type Gn reactivity by normalizing to the OD450 nm/630 nm of wild-type Gn. Mutations were considered critical to the neutralizing epitopes if they did not support reactivity of the neutralizing antibodies.

### Attachment inhibition assay

Vero-E6 cells were cultured in 24-wells plate (Corning) and treated with 50 nM Bafilomycin A1 (Selleck) at 37 °C for 1 h. At the same time, 2 × 10^4^ TCID_50_ rMP-12 was co-incubated with 100 μg or 10 μg A38 or A13 antibody at 37 °C for 1 h. Then the virus-nAbs mixtures were added to the Bafilomycin A1 treated cells and incubated at 37 °C for 1 h. After three times wash to remove the unbound virions, the cells were analyzed for viral RNA loads by qRT-PCR.

### Membrane fusion inhibition assay

Vero E6 cells were infected with Ad5-GnGc (MOI = 10) for 12 h, then 10^4^ infected and 10^4^ uninfected Vero E6 cells were co-cultured in 96-well plate for 24 h. Following incubation with 10 μg or 30 μg A38 or A13 antibody at 37 °C for 1 h, the cells were treated with mildly acidic PBS (pH 5.3) at 37 °C for 20 min and cultured at 37 °C for 5 h. Then the cells were fixed with 4% paraformaldehyde at room temperature for 1 h and incubated with anti-Gn monoclonal antibody E2 (1 μg /ml) at 37 °C for 1 h. After three times wash with PBS, FITC labelled goat anti-human IgG Fc (Abcam, ab97224, 1:1000) was added to the microplates and incubated at 37 °C for 1 h. Washed again, the cells were stained with DAPI (Thermo Fisher Scientific, 1:1000) and the syncytia were analyzed by High Content Analysis System (PerkinElmer).

### Antibody structure prediction

The structures of antibody variable regions were predicted by ABodyBuilder2 [36] with default parameters, using paired antibody chains (heavy and light) as input for A13 and A38. Then, the “Annotate Antibody Sequence” tool in BIOVIA Discovery Studio version 4.5 was employed to annotate the sequences of the antibody variable regions. Subsequently, according to the Chothia annotation scheme [52], the residues in the antibody structures were renumbered.

### Antibody – antigen docking and structure analysis

The ClusPro web server [37] (https://cluspro.org) was used in antibody mode with default settings, providing automated masking for the non-CDR regions of antibodies. ClusPro generates multiple docking poses of the antibody–antigen complex, outputs the docking poses at the centres of the 10 most populated clusters and ranks the models by cluster size.

The interface of the antigen–antibody interaction was analyzed using the “Structure Analysis (H-Bonds)” tool in UCSF ChimeraX version 1.2 [53] and Ligplot + [54]. The superposition of different protein complex structures was performed using the “Structure Analysis (Matchmaker)” tool in UCSF ChimeraX version 1.2.

## Author contributions

Meng Hao and Ting Bian conducted the majority of the experiments, analyzed the results, and drafted the manuscript. Zhengshan Chen assessed the genetic characteristics of bAbs and nAbs, predicted the antibody structures, and conducted antibody – antigen docking as well as structural analyses. Chuanyi Zhao and Guangcheng Fu assisted with protein purification. Yi Chen provided support for the animal experiments. Xiangyang Chi, Pengfei Fan, and Guanying Zhang contributed to antibody screening. Ting Fang assisted with reagent procurement. Changming Yu and Jianmin Li supervised the entire study, reviewed and revised the manuscript. All authors contributed to the article and approved the submitted version. Meng Hao, Ting Bian and Zhengshan Chen contributed equally to this manuscript.

## Supplementary Material

Supplementary_materials-clean.docx

## Data Availability

All data that support the findings of this study are available from the corresponding author upon reasonable request.
