# Supplementary material for: Potent neutralization of Rift Valley fever virus mediated by monoclonal antibodies via concurrent inhibition of attachment and fusion
Source: Emerg Microbes Infect. 2026 Feb 10;15(1):2623698. doi: 10.1080/22221751.2026.2623698 (PMC12893178; doi:10.1080/22221751.2026.2623698)
Supplement: Supplementary_materials-clean.docx [file TEMI_A_2623698_SM1218.docx]

**Supplementary Materials**


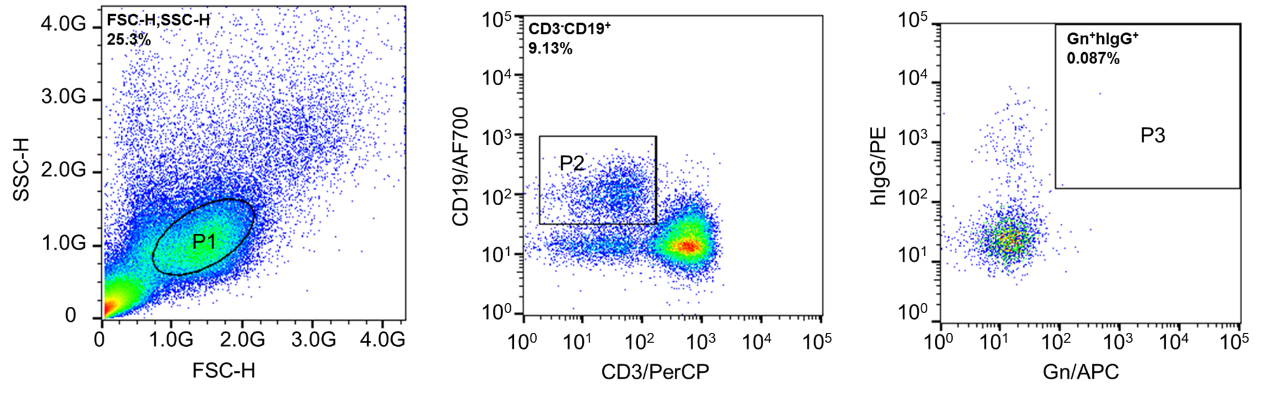


**Supplementary Figure 1.** Gating strategy for antigen-specific memory B cells.

**
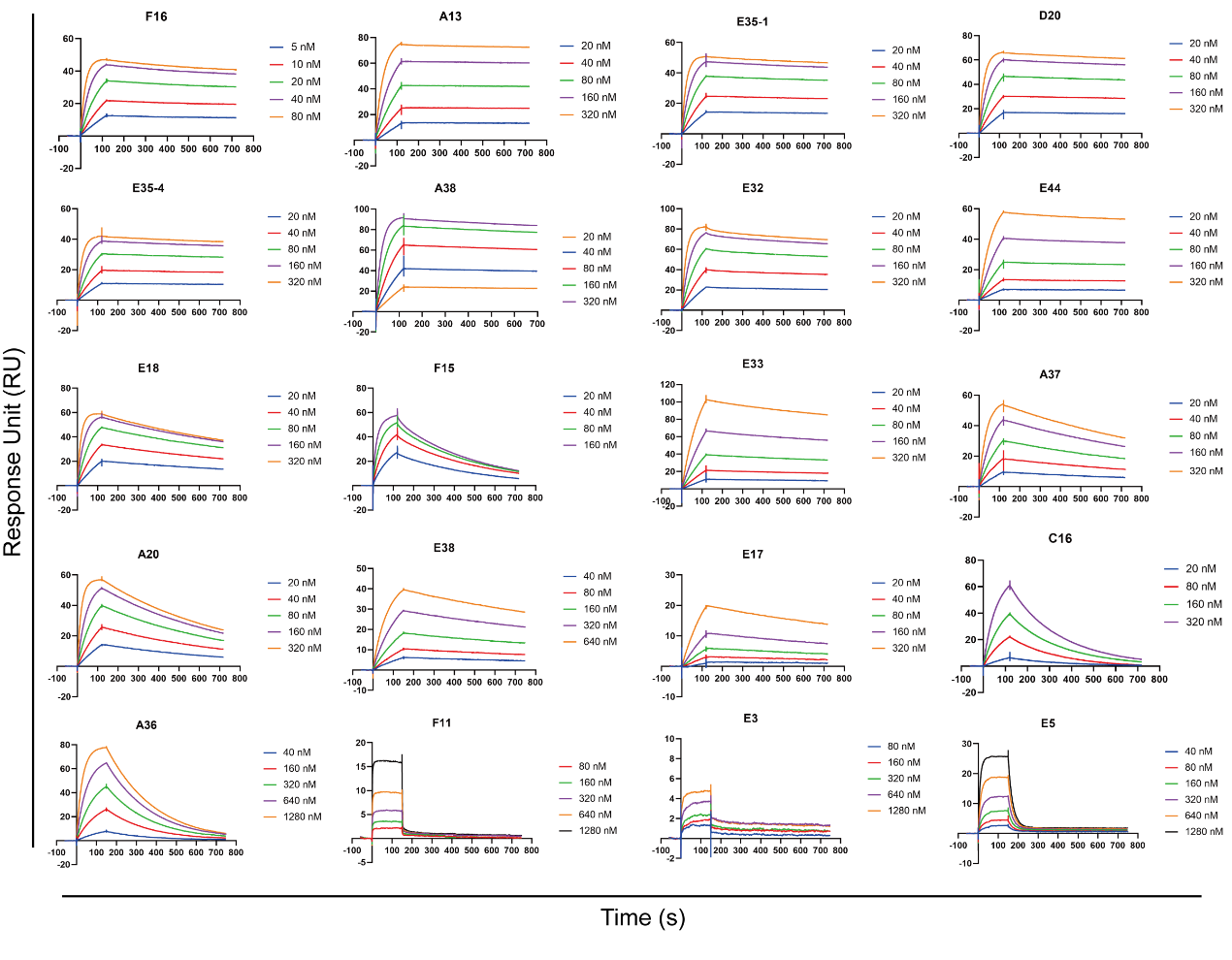
**

**Supplementary Figure 2.** The binding kinetics of indicated monoclonal antibodies to RVFV Gn protein were analyzed by SPR. nAbs were diluted with HBS-EP+ to a concentration of 0.5 μg/mL and loaded to Protein A chip at flow rate of 10 μL/min for 60 s. Then the Gn protein was serially two-fold diluted, combined with nAbs on chip at flow rate of 10 μL/min for 120 s and dissociated for 600 s at same flow rate. The association and dissociation sensorgrams are shown.


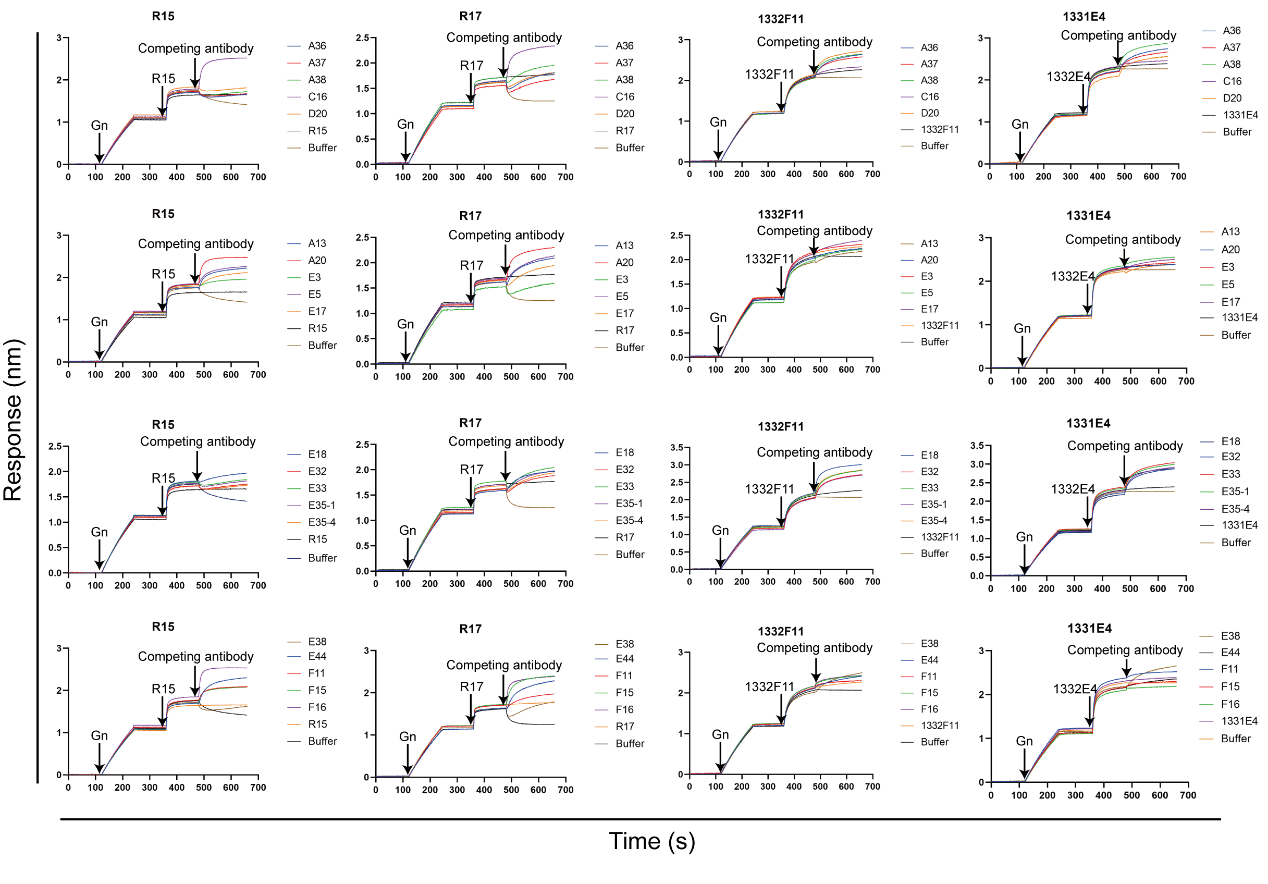


**Supplementary Figure 3.** The competitive binding properties between neutralizing antibodies and representative antibodies were analyzed by BLI competition assay. The biotinylated Gn protein was diluted to 100 nM and loaded onto the streptavidin biosensors for 120 s at 400 rpm prior to baseline equilibration for 120 s. Association of representative antibodies (R15, R17, 1332F11 and 1331E4) at 300 nM was performed for 120 s prior to association of competing antibodies at 300 nM for 240 s. The association and dissociation sensorgrams are shown.

**
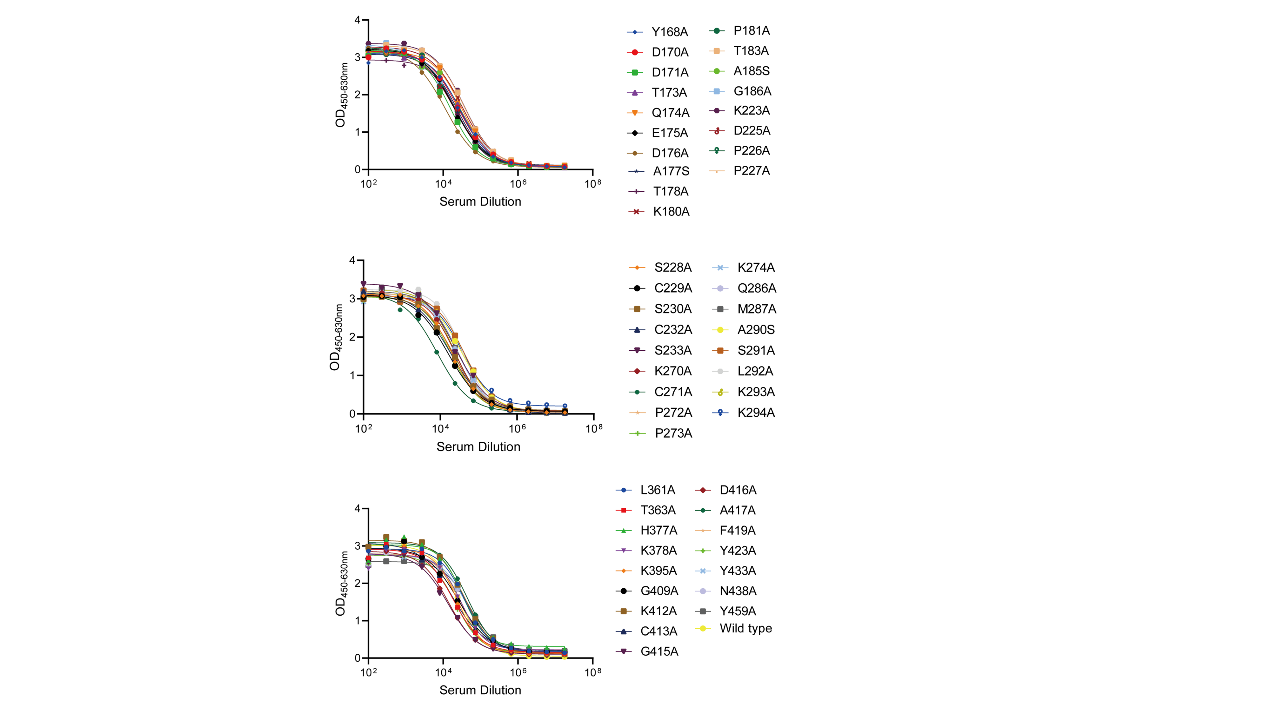
**

**Supplementary Figure 4.** Binding activities of Gn protein mutants to anti-Gn rabbit polyclonal antibody were measured by ELISA.


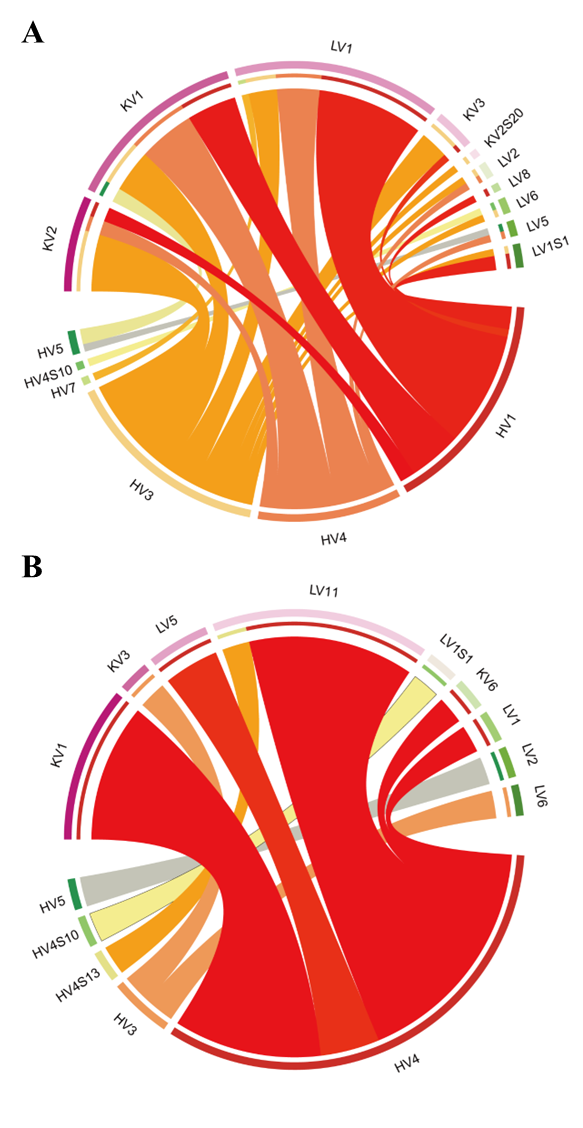


**Supplementary Figure 5.** Combination of heavy chain and light chain of Gn protein-specific antibodies (A) and RVFV neutralizing antibodies (B). Each arc of the circle represents a family of IG VH and VL gene. The bands between two families represent the combination of VH and VL genes. The width of bands represents the frequency of combination.


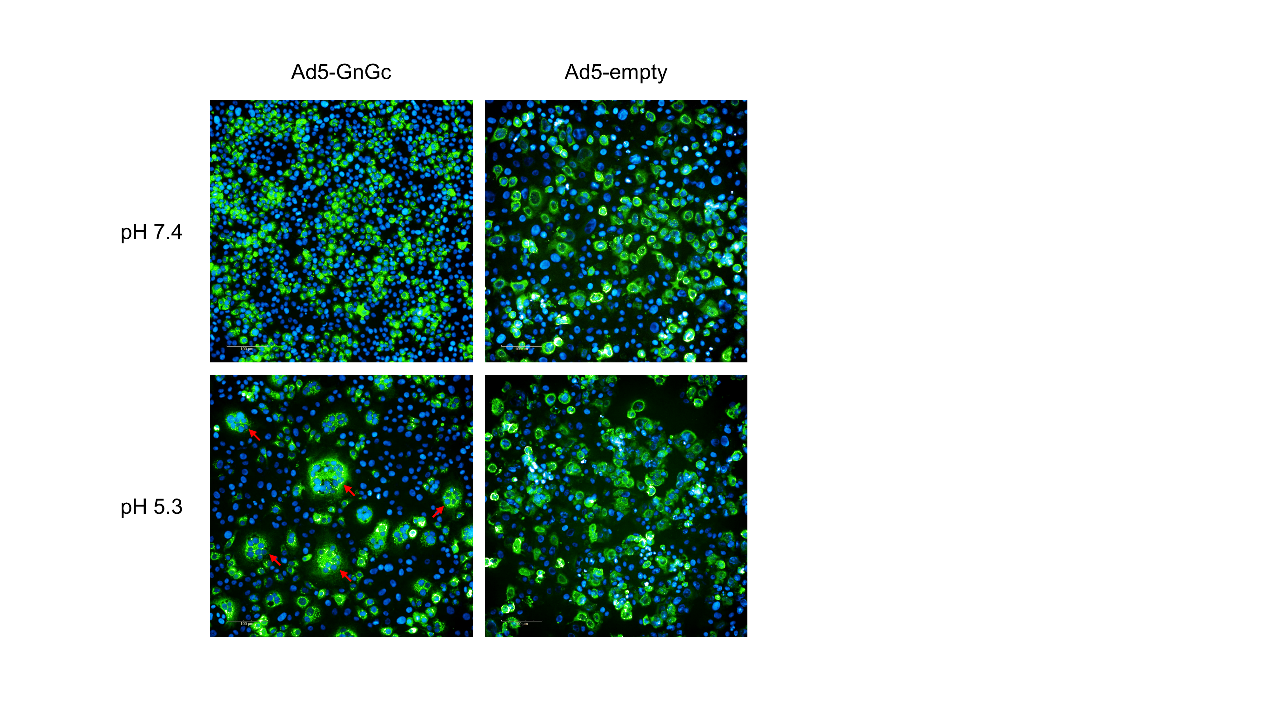


**Supplementary Figure 6.** Low pH-triggered syncytia formation of Ad5-GnGc infected cells. Vero E6 cells were infected with Ad5-GnGc at MOI of 10 for 12 h, then the infected and uninfected Vero E6 cells were co-cultured in 96-well plate in a 1:1 ratio for 24 h. After that, cells were treated with mildly acidic PBS (pH 5.3) at 37 °C for 20 min and cultured at 37 °C for 5 h. Cells were fixed, Gn protein was staining with specific monoclonal antibodies (Green), and nuclei were stained with DAPI (blue). The red arrow represents the syncytium.

**
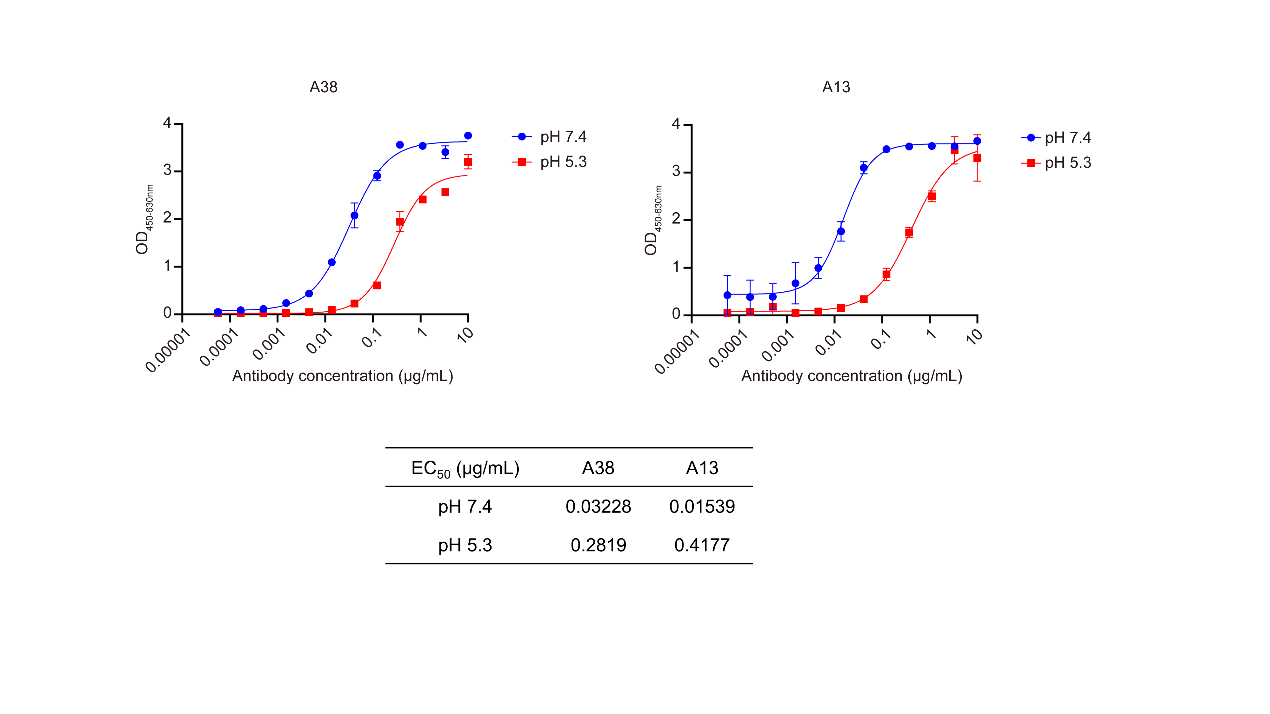
**

**Supplementary Figure 7.** Binding activity of A38 and A13 to Gn protein in pH 7.4 and pH 5.3 conditions were tested by ELISA.


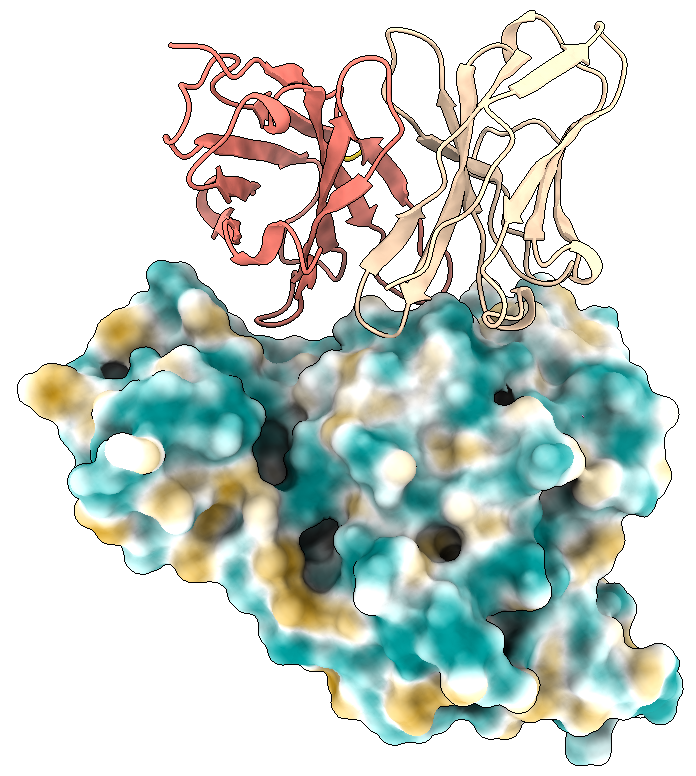

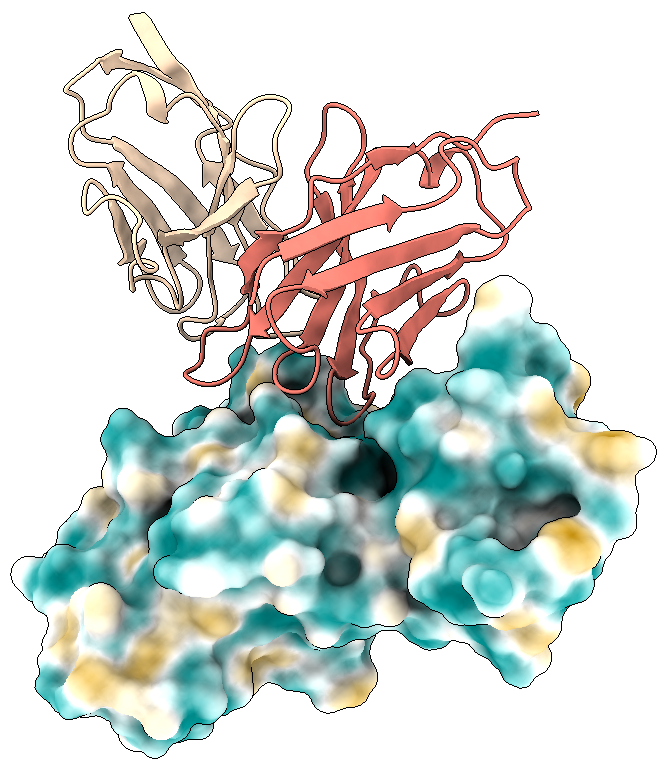


**Supplementary Figure 8.** Hydrophobic interactions between the variable regions of A38 (left) and A13 (right) with the Gn head domain surface. HC and LC were shown as cartoons and colored red and pale yellow, respectively. The Gn head domain surface was colored according to hydrophobicity, with orange to cyan indicating hydrophobic to hydrophilic.
